# Generative Adversarial Networks for Morphological–Temporal Classification of Stem Cell Images

**DOI:** 10.3390/s22010206

**Published:** 2021-12-29

**Authors:** Adam Witmer, Bir Bhanu

**Affiliations:** 1 Visualization and Intelligent Systems Laboratory, University of California, Riverside, CA 92521, USA; bhanu@ee.ucr.edu; 2 Department of Bioengineering, University of California, Riverside, CA 92521, USA; 3 Department of Electrical and Computer Engineering, University of California, Riverside, CA 92521, USA

**Keywords:** dataset augmentation, deep learning, developmental toxicology, generative adversarial networks, stem cell biology

## Abstract

Frequently, neural network training involving biological images suffers from a lack of data, resulting in inefficient network learning. This issue stems from limitations in terms of time, resources, and difficulty in cellular experimentation and data collection. For example, when performing experimental analysis, it may be necessary for the researcher to use most of their data for testing, as opposed to model training. Therefore, the goal of this paper is to perform dataset augmentation using generative adversarial networks (GAN) to increase the classification accuracy of deep convolutional neural networks (CNN) trained on induced pluripotent stem cell microscopy images. The main challenges are: 1. modeling complex data using GAN and 2. training neural networks on augmented datasets that contain generated data. To address these challenges, a temporally constrained, hierarchical classification scheme that exploits domain knowledge is employed for model learning. First, image patches of cell colonies from gray-scale microscopy images are generated using GAN, and then these images are added to the real dataset and used to address class imbalances at multiple stages of training. Overall, a 2% increase in both true positive rate and F1-score is observed using this method as compared to a straightforward, imbalanced classification network, with some greater improvements on a classwise basis. This work demonstrates that synergistic model design involving domain knowledge is key for biological image analysis and improves model learning in high-throughput scenarios.

## 1. Introduction

Stem cells are unspecialized cells that are used as a model for early-stage growth. They recapitulate biological characteristics of embryonic development, most importantly pluripotency, or the lack of specified cellular purpose [1]. Deviations from this pluripotent state are an indication of differentiation, or phenotypic lineage commitment, and have implications on the health and developmental status of cells and cellular colonies [2].

The normal growth and downstream differentiation cycles of pluripotent stem cells are highly coordinated and delicate processes. Much work has been performed to delineate and manipulate these molecular changes in vitro in order to better understand the mechanisms by which they occur [3,4,5]. For example, adult cells have been turned back into stem cells in vitro (induced pluripotent stem cells, iPSC’s), and normal stem cell differentiation has been modeled using Markovian stochastic methods [6,7,8].

These and other studies have determined that stem cells transition from the pluripotent state to the differentiated state via an intermediate progenitor and that many unobservable substates exist within these larger, observable phenotypes. These phenotypes present themselves with distinct morphological structure and can be observed via light microscopy as cellular colonies with unique gray-level texture patterns. Depending on the duration of in vitro differentiation, varying proportions of cellular colonies at given stages are observable at different points in time.

**Figure 1:**
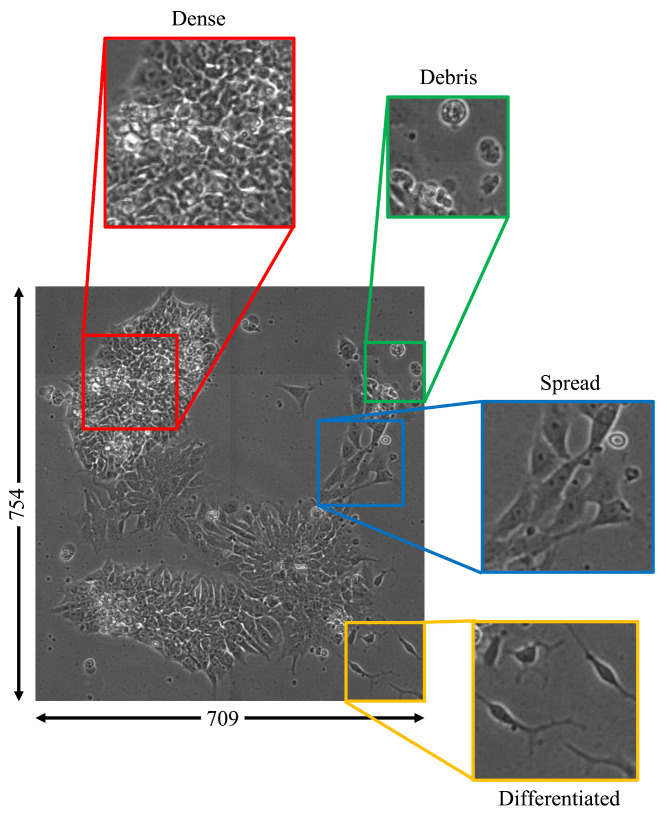
Image examples for four morphological classes observable in a single cell colony (debris: green; dense: red; spread: blue; differentiated: yellow). Throughout the differentiation process, various proportions of each class can be found in cell colonies with contiguous cell boundaries. Classification of these multiclass images can be performed using image patches.

For example, in the beginning of the process, there are more of the early-stage cell class, while in the middle all three stages can be observed, and there may be some of the late-stage, and more of the intermediate stage. Toward the end of this cycle, given a high yield, there should be more of the fully differentiated stage than the earlier two classes. Figure 1 displays the nature of the multiclass cell colonies with contiguous cell boundaries that result in the four morphological classes used in this paper (debris, dense, differentiated, and spread) as described in Table 1. The spatiotemporal way data are collected over the course of this process provides snapshots of each of these stages. The normal differentiation process is subject to both internal cues and external factors, and the balance of these signals can influence cellular fate. Stem cells are particularly susceptible to external perturbations, and early molecular changes such as DNA mutations can have long-term effects on cellular and organismal health.

### 1.1. Developmental Toxicology

Developmental toxicology is the study of the effects of environmental factors on prenatal growth and development [9]. In vitro studies aimed at observing the developmental effects of exposure to tobacco chemicals on stem cells have been influential in the formation of United States Food and Drug Administration (FDA) policies for harm reduction and public health. However, the pace of stem cell research is often limited by the tedious and time-consuming process of image data analysis. In addition, the desired scale of experiments involving the testing of multiple chemical compounds at various concentrations, across multiple cell types, in a high-throughput manner, results in data that are often impossible to analyze by hand. Recently, computational analysis using video bioinformatics has become an invaluable tool for researchers toward reducing human error and increasing analytical throughput.

### 1.2. Video Bioinformatics and Machine Learning

According to Bhanu et al. [10], video bioinformatics (VBI) is “the automated processing, analysis, understanding, data mining, visualization, query-based retrieval/storage of biological spatiotemporal events/data and knowledge extracted from videos obtained with spatial resolution varying from nanometer to meter of scale and temporal resolution varying from seconds to days and months." Many aspects of experimentation can be quantified by observing cellular behavior in images and videos in a noninvasive manner (i.e., without killing or otherwise perturbing live cells). Light microscopy is often used to observe dynamic colony behavior by collecting time-lapse images during experimentation. Data collection is sometimes automated using an incubator–microscope unit such as the Nikon Biostation CT to accumulate temporal image data [11]. These units are programmed to collect whole-dish images, or perform single colony tracking at desired time intervals, for extended periods of time, helping to standardize data collection in a high-throughput manner.

A bottleneck arises in the analysis of resulting image data, normally processed by hand over the course of many weeks, or with the aid of open source software such as ImageJ and CLQuant [12,13]. These rudimentary programs require users to sift through their dataset, image by image, selecting, outlining/tracing, and visualizing images or creating image processing pipelines designed to generalize across the dataset. These algorithms are useful tools for segmenting and measuring objects in an image but require substantial expertise and user input, which comes with the possibility of increased error due to nonstandardized bias.

In the past decade, many advances have been made to remove user error and bias by automating the image analysis process. The utility of VBI programs has expanded to include quantification and classification of results. In general, these image processing programs are concerned with leveraging unique characteristics (i.e., features) of images, at both the pixel and image level, to accomplish a desired task, such as classification. Some examples of features include global static information such as colony size, shape, and morphology, as well as temporal information including motility, growth rate, and observed behavioral status (e.g., differentiation or death) [14]; local features include information found within patterns, such as texture, contrast, intensity, and color. Programs that exploit these features are useful to researchers for removing sources of human error by combining, standardizing, and automating the feature extraction and quantification/classification processes. For example, Guan et al. use a Gaussian mixture model to segment stem cells from image background in static microscopy images [15].

Many of these programs also employ machine learning algorithms such as clustering, decision trees, or support vector machines to improve classification. One such program, called StemcellQC, analyzes time-lapse microscopy videos using predetermined, hand-crafted morphological features of stem cell colonies. This program takes input from the user via a graphical user interface (GUI) in terms of setup and desired output, and automatically analyzes and plots outputs for the user to view [14]. Global features such as colony area, aspect ratio, and motility are combined with local features, including gray level intensity, to classify individual colonies by health status (healthy, unhealthy, dying) using standard machine learning algorithms to determine the effects of toxic chemicals on cellular behavior.

Another program, Pluri-IQ uses a supervised random forest classifier to distinguish between cell colonies at different stages of growth from pluripotent to differentiated in dense, fluorescent microscopy images [16]. While these software improve the efficiency of analysis through standardization, they still require user interaction, rely on the researcher to predetermine features based on prior knowledge of colony behavior, or exploit some previously observed pattern. More recently, deep learning has revolutionized image analysis by automating both the feature extraction and classification processes to remove sources of human error and bias. The following section discusses deep learning approaches for biological image analysis.

### 1.3. Deep Learning Approaches

Deep learning (DL) programs help to overcome the drawbacks of data analysis by combining feature extraction and classification into a single model. DL models do this by determining mathematical features of images that can be used to categorize input data. These features consider all aspects of an input image and are iteratively refined with respect to a desired output using a gradient descent optimization algorithm. Moreover, deep learning extends these algorithms to image processing and analysis via deep convolutional neural networks (CNN). The term deep comes from the layered architecture of the CNN; neural network comes from the weighted connections between a pair of layers, and a bioinspired gated activation operation, the rectified linear unit (ReLU) is like the all-or-nothing activation response of neurons to an input signal [17]:

(1) $$f_{node}=\sum_{i=1}^{n}\left(w_{i}x_{i}\right)+b$$

(2) $${\mathrm{ReLU}}_{y}=\left\{\begin{array}{cc} y=0 & y\leq 0\\ y=y & y>0 \end{array}\right.$$

These algorithms sequentially multiply input images by the layered weights as shown in Equation (1). At each node, the output is $f_{node}$, $x_{i}$ is the input value from the previous layer, $w_{i}$ is the weight value of the layer, and *b* is an additional bias term. Positive valued outputs are fed forward to the next layer through a piecewise ReLU activation function (Equation (2)), where *y* is the feature map from the previous layer. The output of these operations at the end of the network is a unique numerical signature that is used to determine the class of the image.

During training, predictions of the network are used to update the parameters (weights) of the model with respect to a given ground-truth. While DL was initially employed to classify extremely large, real world datasets such as ImageNet [18], it has been used recently to improve the accuracy and efficiency of analysis for biomedical applications including microscopy [19,20,21,22], high-throughput methods [23], MRI [24], histopathology [25], and stem cell microscopy imaging [26]. For example, in our previous work, the patch-based classification of multilabel colony images was addressed in [27]. However, the aforementioned caveat of dataset size, as well as the large size (224 × 224) of image patches used in this work were noted as drawbacks to this method.

The high-parameter nature of these networks requires training them with extremely large datasets to avoid overfitting, which is the case where the model learns to classify the training images perfectly, instead of learning features that generalize well across the testing dataset. There is commonly a positive correlation between dataset size and network accuracy, because the more sample images the network sees during training, the more precisely it models the data and, consequently, the more information it uses to make decisions during testing. Unfortunately, the nature of biological experimentation frequently limits the size of the dataset, based on time, resources, and general difficulty in performing experiments.

Therefore, there is a need to increase the size of biological datasets by supplementing images with similar and visually relevant data, without having to perform new experiments to collect more images. Generative adversarial networks (GAN) provide a unique opportunity to generate new images that are representative of the real data. This paper is aimed at performing data augmentation by supplementing a minimal biological dataset via image generation using GAN [28]. GAN are a subset of DL networks that combine two opposing networks that take a noise vector as input and produce an image based on the features that they learn from the real dataset. More information on these complex networks is provided below.

### 1.4. Generative Adversarial Learning

GANs are DL models that combine two networks, a generator (*G*) and a discriminator (*D*), that play a min-max learning game to model input data (Equation (3)). *G* takes as input a vector, *z*, of numbers sampled from a Gaussian distribution, and performs upsampling convolutions to produce and n×n size image, *G(z)*. *D* alternately takes as input either real or generated images and performs downconvolutions to produce a realness score that is used to determine if the image is real or fake. The goal of *G* is to generate images that fool *D* into thinking that they are real (i.e., minimize the probability that *G* comes from the fake distribution, *p_z_*), and the goal of *D* is to maximize the log-likelihood probability that a given image comes from the real distribution *p_data(x)_*, where E is the expected value operation:

(3) $$\underset{G}{min}\;\underset{D}{max}\;V\left(D,G\right)\;=E_{x∼p_{\mathrm{data}(x)}}\left[\log\;D\left(x\right)\right]+\;E_{z∼p_{z}\left(z\right)}\left[\log\left(1-D\left(G\left(z\right)\right)\right)\right]$$

GANs learn a representation of input features in an unsupervised manner, which can then be used for additional downstream learning tasks such as feature extraction and classification [29]. Convolutional GANs were originally designed to generate images from extremely large, open-source datasets, such as natural images (Imagenet [17], CIFAR [30]), faces, and numbers (Mnist, [31]). More recently, this work has been expanded into unique datasets including those from medicine and biological experimentation. Efforts to address the challenges of modeling a unique cellular microscopy dataset using GAN, and the use of generated data for dataset augmentation, are the subject of this work. Related works involving GAN are presented in the following section.

## 2. Related Works

Recently, GANs have been used for biological image generation tasks involving cellular microscopy and medical image datasets [32,33]. For the purposes of this paper, cellular microscopy images are considered distinct from medical images (e.g., X-ray, CT, MRI) in that they deal with objects on a micrometer scale and are usually obtained from cellular experimentation involving microscopy. Factors including scale and cellular morphology increase the visual complexity of data and, in turn, that of modeling/analysis. There are many ways to implement GANs for learning applications using cellular image data with the goal of gathering useful features in an unsupervised manner. Some examples of cellular microscopy applications of GAN are given below.

One indirect method of using GAN features is through transfer learning, in which the information learned from GAN, in the form of network parameters, are leveraged by separate models to improve network performance when using few labeled data. For example, Majurski et al. [34] use features from GAN trained on fluorescent stem cell microscopy images to perform cellular segmentation with a U-net style pixelwise classification network. Wang et al. [35] use GAN discriminator features to fine-tune a classifier on minimal labeled dataset for detection of rosette formation in early stage *C. elegans* embryos. While transfer learning using GAN can indirectly inform downstream model learning, GAN can also be used to directly produce image outputs for observation of the learned representation.

An example of this is the task of image-to-image translation, in which the original style or modality of an image is transformed to another of desired nature. Rivenson et al. [36] used a VAE-GAN style network, in which an encoder–decoder–discriminator network is used to translate images acquired using a noninvasive phase contrast microscopy, into colorized histology images. This digital staining technique is used to circumvent the difficult process of histology staining, in favor of noninvasive microscopy techniques. Lee et al. [37] perform 3D fluorescent microscopy deconvolution using a specialized CycleGAN framework. This network performs image-to-image style transfer using multiple GANs to learn a mapping between unpaired images of different styles, such that there is no need for a direct ground-truth comparison during training. They use this framework to sharpen noisy images for improved segmentation in 3D volumes of rat kidney sections. Bailo et al. [38] generate images of red blood cell smears to perform dataset augmentation for segmentation and detection tasks. They train a sophisticated image generator (pix2pix) to perform image-to-image translation from segmentation masks of real images and subsequently generate new images from synthesized segmentation masks. These works utilize GAN to change images from one style or modality to another but do not directly employ GAN for image generation from a latent space.

More straight forward implementations of GAN involve the use of networks to directly generate images from a learned feature representation using only a latent variable as input. Goldsborough et al. [39] generate single cell fluorescent images in three color channels using various GAN models and perform image interpolation before using GAN features for transfer learning, observing increased classifier performance. Pandhe et al. [40] combine a GAN image representation with autoregressive motion synthesis to accurately recapitulate neutrophil behavior and observe patterns of organelle function. Theagarajan et al. [41] generate single cell images of human embryonic stem cells across five health-related classes using multiple networks. They use an ensemble of GAN networks to generate thousands of stem cell images for dataset augmentation and find increases in evaluation metrics for the number of added images. Osokin et al. [42] use a “separable generator” to generate two color channels of a multichannel fluorescent images. In all these cases, the generated images are of whole cells, where the entire cell body is within the field of view. Images like these are generally less difficult to model than the more detailed, varied, and fine-grained texture patterns observed in the image patches generated in this work, because the model can learn the relationship between the background and foreground.

The work of Devan et. al. [43] provides another example of a GAN implementation for limited biological dataset augmentation. The authors of this work use GAN to increase the size of a transmission electron microscope image dataset. They perform automatic detection of cytoplasmic capsids using region-based CNN (R-CNN) [44] with an augmented dataset and show an improvement of their results against the standard dataset configuration. This method employs SinGAN [45] to generate alternative versions of real images using a pyramidal GAN network. Unlike this work, the proposed method seeks to use GAN to generate completely new image patches to be added to the dataset, instead of different versions of real images. Furthermore, the proposed method also takes class relationships and dataset imbalances caused by experimentation into account.

Similarly, Dimitrakopoulos et. al. [46] perform GAN-based dataset augmentation for open-source medical image datasets. They propose a GAN model, Ising-ResGAN, that uses Markov random field constraints to perform image smoothing. They use generated images to improve the results of a U-net segmentation task for various publicly available datasets. They generate images with a large field of view (256 × 256) and do not include domain specific knowledge, where as the proposed method generates subcolony image patches and incorporates a learning scheme based on stem cell differentiation.

### Contributions of this Paper

While the above related work represents useful applications of GAN to biological datasets, none of them addresses the issue of temporally constrained differentiation. The previously mentioned works involve the modeling of static images with varying levels of image complexity in terms of cellular structure and colony density, texture, and overall variation across datasets. These works do not impose biological constraints or exploit domain knowledge. In this paper, a mathematical model of stem cell differentiation, involving Markov chain stochastic processes, is used to inform model training.

The focus of the proposed approach is on the generation of small image patches containing fine grained texture features and high variation, across four classes (dense, spread, differentiated, and debris) using GAN. This paper expands significantly on previous work in [47] by testing multiple model configurations/architectures and introducing a new GAN training/quality control scheme that utilizes image entropy distributions for improved training. These aspects of the proposed model make it novel in comparison to the previous work. The contributions of this paper are as follows:

(1) Models complex, varied, and highly textured image patches using GAN
(2) Incorporates domain knowledge in the form of temporal constraints on model learning as well as bioinspired algorithm design
(3) Introduces an image-entropy-based metric for model training, image postprocessing, and quality control
(4) Explores dataset augmentation as a viable means for improving network performance for tasks involving patch-based classification

The data used in this work present unique challenges in terms of image generation and classification. Specifics of the proposed method to address these challenges are discussed in the following section.

## 3. Materials and Methods

### 3.1. Technical Approach

**Figure 2:**
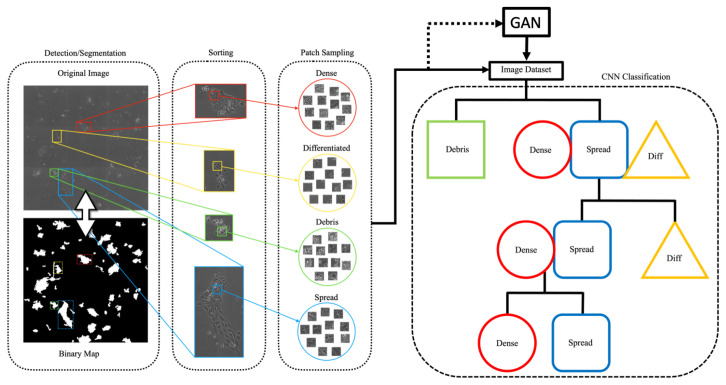
Data preprocessing and classification schematic. The binary map of colony locations is used to segment colonies from the original image, which are then sorted by hand during ground-truth generation (**left**). Patches from the resulting dataset are used to train the GAN. Generated images are added to balance the dataset for the temporal CNN classification scheme (**right**), during which images are sorted into their individual classes through multiple hierarchical stages.

Figure 2 describes the overall approach for colony detection and image preprocessing used in this work. The preprocessing step is performed to reduce the amount of flat background in images, which contains no class-relevant information. The large size images from this dataset are too computationally expensive to be processed as whole images, and relevant colony areas make up only a portion of the original stitched microscope image. Cell colonies are detected using a morphological segmentation algorithm (sequential operations: 3 × 3 Gaussian blur, entropy filtering (disk filter, size 3), morphological opening (disk filter size 3), binarization via Otsu thresholding, hole filling, small object removal <2000 pixels). Detected colonies are cropped out to amass a dataset of colony image ROIs. After this, random patches of cropped images are used to train separate GAN models for each class.

The proposed method employs GAN to model four separate data classes (dense, spread, debris, differentiated; see Table 1). These classes are determined by the morphological appearance of cell colonies in static images and correspond to cellular phenotypes, or specific cell types, based on prior biological knowledge. The use of multiple GAN networks (one for each class) in this work has several advantages including: 1. negating the effects of class imbalances; 2. improved training because of modeling a unimodal distribution of class features; 3. allowing for the specific tailoring of each GAN for a single class (i.e., number of training epochs for convergence); 4. feature disentanglement via extraction of classwise information, which includes the entropy loss calculated during training.

### 3.2. GAN Architectures

In this work, several generative adversarial network (GAN) architectures and loss functions are tested to determine the most advantageous configuration for the specific task and dataset. Given that GAN training is notoriously unstable, a deep convolutional GAN (dcGAN) [29] architecture is adopted as a base model (Table 2). The GAN generator, *G*, takes as input a 100-dimensional Gaussian noise vector and outputs a 64 × 64 grayscale image. That image is then fed alternately with real images to the discriminator, *D*. *D* then outputs an adversarial (real/fake) score for the image, which is the binary cross-entropy criterion, where *L* is the loss value, *x* is the network output with respect to the input *c*, and the score is computed across all samples, *j* Equation (4):

(4) $$L\left(x,c\right)=-x\left(c\right)+\log\left(\sum_{j}e^{x_{j}}\right)$$

(5) $$L_{\mathrm{dcGA}N_{\mathrm{Gen}}}=L_{\mathrm{Adversaria}l_{P_{z}}}$$

(6) $$L_{\mathrm{dcGA}N_{\mathrm{Dis}}}=L_{\mathrm{Adversaria}l_{P_{z}}}+L_{\mathrm{Adversaria}l_{P_{\mathrm{data}}}}$$

The standard loss function for the generator is the adversarial loss with respect to the generated data distribution, $P_{z}$, (Equation (5)). The aggregate loss function for the discriminator is the average of the adversarial losses for both the generated, $P_{z}$, and real image samples, $P_{\mathrm{data}}$, (Equation (6)). GAN training uses the Adam optimization algorithm along with the parameters provided in Table 3. These learning values are determined empirically using network optimization and allow for stable, efficient training of the GAN.

Several other GAN models and loss functions are compared to this baseline to determine the effect of GAN configuration on generated image quality. These architectures include the Wasserstein GAN (wGAN) [48], Auxiliary GAN (auxGAN) [49], and Metropolis-Hastings GAN (mhGAN) [50]. Each of these networks use the dcGAN as a framework on which to build specific training/learning techniques and loss functions. Brief overviews of each configuration are outlined below.

#### 3.2.1. Wasserstein GAN

The Wasserstein GAN (wGAN) is a network configuration that tries to solve the problem of “vanishing gradients" in normal GAN applications that lead to the phenomenon of mode collapse in image generation. Mode collapse is when the generator fails to model all the variability in the input dataset and instead learns to output images that contain only a small subset of input features. This is often due to the discriminator learning a good mapping between real and fake images, which prevents the generator from training efficiently. wGAN attempts to control the weights of the discriminator by restricting, or “clipping”, the highest and lowest weights within the discriminator feature maps to allow the generator to learn a more sufficient mapping of the dataset distribution during training.

#### 3.2.2. Auxiliary GAN

The auxiliary GAN is a dcGAN network that uses image labels as a condition for network training. The image labels are provided to the generator network as a latent embedding, which gives the network prior information about image class. The generated images are then passed to the discriminator, which outputs both an adversarial score, as well as an auxiliary classification score in the form of cross-entropy loss criterion (softmax function + negative log-likelihood). The loss function for this network then becomes the aggregate of the adversarial and auxiliary losses for both the generator and discriminator, as shown below in Equations (7) and (8):

(7) $$L_{\mathrm{auxGA}N_{\mathrm{Gen}}}=L_{\mathrm{Adversaria}l_{P_{z}}}+L_{\mathrm{Auxiliar}y_{P_{z}}}\;$$

(8) $$L_{\mathrm{auxGA}N_{\mathrm{Dis}}}=L_{\mathrm{Adversaria}l_{P_{z}}}+L_{\mathrm{Adversaria}l_{P_{\mathrm{data}}}}+L_{\mathrm{Auxiliar}y_{P_{z}}}+L_{\mathrm{Auxiliar}y_{P_{\mathrm{data}}}}$$

The advantages of the auxGAN configuration are that it allows multiple image classes to be produced by the same generator. However, the network has the more difficult task of modeling a multimodal distribution, which could affect individual classwise image quality.

#### 3.2.3. Metropolis-Hastings GAN

The Metropolis-Hastings generative adversarial network (mhGAN) is a dcGAN implementation that uses Markov chain Monte Carlo sampling to try to improve image generation. mhGAN attempts to find a more accurate image representation for the generator using the discriminator to guide image selection via the Metropolis-Hastings algorithm. Equation (9) illustrates how the model is able to learn the relationship between the data distributions, pD(x) and pG(x) using the output of the discriminator, D(x). The discriminator is provided multiple real and generated samples and is tasked with determining the most relevant real images with which to train the generator, based on its decision function:

(9) $$\frac{p_{D}\left(x\right)}{p_{G}\left(x\right)}=\frac{D(x)}{1-D(x)}$$

An auxiliary implementation of the mhGAN is also trained here to test the ability of the Metropolis-Hastings algorithm to model the multimodal distribution. All GAN networks are trained to convergence using the Adam optimizer, and training is monitored via loss function and generated image appearance. A table of network hyperparameters for training is provided in Table 3, where all values were empirically determined for optimization. Networks are trained using NVIDIA GeForce GTX 1080ti GPU’s and programmed using the Pytorch deep learning library [51]. This model also utilizes the advice of GAN Hacks for design and implementation (https://github.com/soumith/ganhacks, accessed on 30 October 2021). The effectiveness of the various GAN methods in generating realistic images is assessed using multiple standard image quality metrics, as well as a novel metric introduced in the following section.

### 3.3. Assessing Generated Image Quality

In this work, several standardized methods of assessing generated image quality are used to compare all these implementations. Quality metrics such as inception score [52] and Frécet inception distance [53] are used, as well as a novel image entropy-based technique that is introduced in this paper, and described in the following section.

#### Image Entropy Distribution

The image generation scheme proposed in this work results in image patches that contain various proportions of foreground/background area where foreground textures are representative of subcolony cellular morphology. The random nature of image patch sampling allows for the network to learn both colony body and boundary areas, which are equally important to the overall classification task when trying to encompass the whole colony area. One method of measuring the accuracy of the generated image distribution is by using image entropy. For this, image entropy is calculated as the Shannon entropy (Equation (10)) of the individual generated image patches:

(10) $$H=-\sum P_{\mathrm{hist}}*\log_{2}P_{\mathrm{hist}}$$

During GAN training, image entropy is calculated for mini batches of real and fake images, and the normalized image entropy probability distributions are used to find an image entropy loss parameter, L_H_. Comparison of distributions is performed using the mean squared error (MSE, Equation (11)), evaluated over *n* samples using the squared difference between measurements *Y* and $\hat{Y}$ and then added to the aggregate loss function of the discriminator:

(11) $$\mathrm{MSE}=\frac{1}{n}\sum_{i=1}^{n}{\left(Y_{i}-\hat{Y_{i}}\right)}^{2}$$

**Figure 3:**
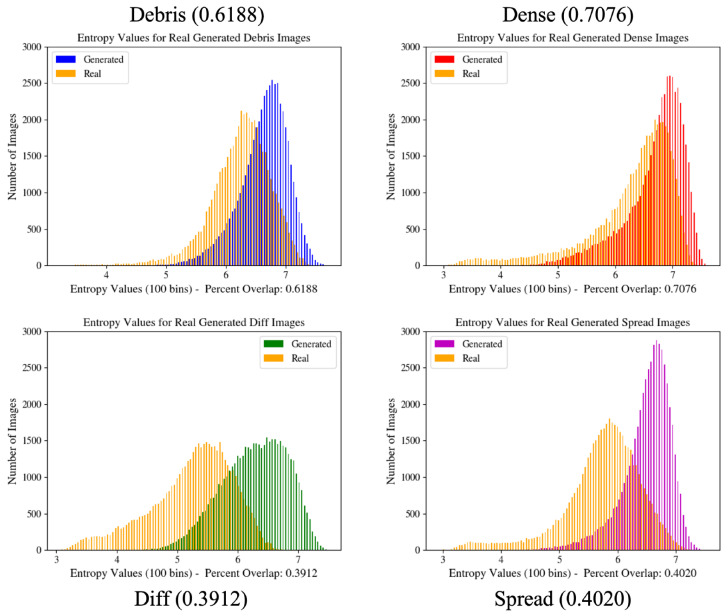
Image entropy distribution histograms for GAN configurations. These graphs provide a quantitative measure of the overall generated image distribution in relation to the real image distribution and are used during GAN training to improve network learning. Values in parentheses indicate the percent overlap of the two graphs shown in the figure.

Trained GANs are used to generate image patches which are added to the real dataset at various percentages and subsequently used to train various CNN models to perform image classification across the four classes. The effect of this added value is observed by plotting the image entropy values of 50,000 random real and generated image patches (Figure 3). The percent overlap between the graphs is calculated by summing the smallest overlapping values across all the bins and dividing by the total number of calculated values. Both the generated and real images have entropy values within the range of 3–8, but the generated images tend to be skewed toward the upper range of this distribution. Higher image entropy is an indication of higher variability in the images and can be interpreted as images with more visual information.

Table 4 shows the average classwise image entropy histogram overlap percentages for five trials of entropy histogram calculations. One trial consists of generating histograms using 50,000 random real and generated image patches each. Random image generation causes variation within these calculations, and is indicative of variability of generated images. These values measure the ability of the generator to model relevant class information and can also be used to inform model learning.

It can reasonably be assumed that the greater the overlap between the real and generated image entropy distributions, the more accurately the generator is able to model the real image patches. These graphs also allow for the visualization of the image distributions in terms of the entropy values and are useful in determining the variability of features that are modeled by the generator. From Table 4, the classes with the lowest overlap values are the differentiated and spread classes. For the differentiated class, this may be due to the relatively small number of images available to the generator for modeling, or the difficulty in modeling features of the specific class. For the spread class, it may be due to the generator learning the high-entropy features of this relatively large class, whereas the real image distribution displays a wider entropy curve.

In addition to calculating a training loss value, this entropy metric is also useful as a measure of the accuracy of the generated image distribution. These values can be correlated to the performance of the downstream data augmentation tasks as well as to the effect of changing the loss function of the discriminator using this entropy criterion as discussed in Section 4 of this paper. The data generated by GAN are then used to augment the real datasets for training classification CNNs with temporally constrained configurations, as described below.

### 3.4. CNN Training Configurations

The main objective of this paper is to improve CNN performance for data limited settings involving biological images. To achieve this goal, the approach used is to augment the real dataset using GAN generated images as described above. However, this task is not as straightforward as it seems. There are many ways in which generated data augmentation can be effective for network training. Two common biological dataset issues are addressed here, namely data imbalances, and limited datasets.

#### Temporal Classification

The dataset configurations employing generated image augmentation are used in conjunction with temporally constrained, hierarchical classification CNNs. Temporal constraints are imposed according to the in vitro differentiation process as modeled by Stumpf, et al. [7]. During this procession, which has been shown to recapitulate in vivo differentiation, cells undergo downstream lineage changes that can be separated into three major categories: embryoniclike stem cells (ESC), intermediate progenitors, and differentiated neurons. Similarly, there are three classes of viable cell colonies that compose the dataset used in this work: dense (ESC), spread (progenitors), and differentiated (neuronlike formations).

**Figure 4:**
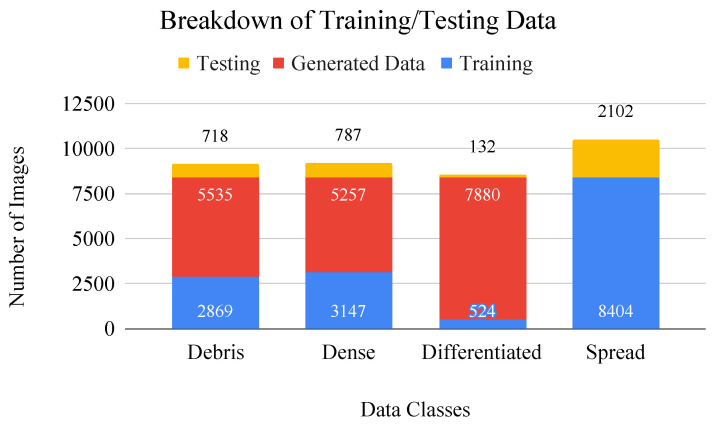
Bar graph of data breakdown including values for training/testing (blue/yellow) split. Generated images (red) are added to the dataset to make up for class imbalances during CNN training.

Additionally, there are colonies of nonviable cells that are known as debris, that represent dead or unhealthy cells and exist independently as well as within viable colonies. These areas are important because they characterize the adverse effects of toxic exposure and provide insight about the health status of cell colonies. Therefore, the hierarchical CNN classification system is set up as a series of two-class networks that combine the various class stages of growth and differentiation as described above. Figure 4 provides a visual reference for data imbalances, as well as the number of images provided for the train:test split. The number of added images in various configurations is shown in relation to the largest class. For example, in the first stage, when dense, spread, and differentiated are combined against debris, the difference between the total number of images in each class is made up by adding generated debris images to the real debris training dataset. At every stage, the smaller, single class is balanced against the larger aggregate class using generated images, creating proportional image classes, in order to counteract the problem of data imbalances.

The temporal constraints imposed in this method focus on the overall morphological class relationships within the dataset. This contrasts with using dynamic cellular changes between video frames directly in model training. Instead, the temporal relationships between image classes are exploited to improve the efficiency of network learning for classification of individual images based on cellular morphology. The details of this method are described below.

First, debris cell colonies are separated from the other three classes, which are grouped together into a single class and sent to the next stage of classification. In the second stage, differentiated cells are separated from a grouped class of dense and spread cells. Finally, in the third stage, dense and spread cells are separated into their individual classes. Performing classification in this manner allows for the exploitation of the natural relationship between classes, as well as allows for more fine control of dataset augmentation using generated images based on class imbalance and dataset proportions.

In the final stage of classification, the power of generated features for dataset augmentation is explored by testing the saturation point for the dense vs. spread classes. Thousands of generated images are added to both the dense and spread classes and a plot of network performance vs. augmentation level is created for visual reference. Empirically, it is shown here that this combination of hierarchical classification and dataset balancing using generated image augmentation outperforms a four-class CNN configuration using various standard dataset balancing methods, and that there is a positive correlation between the level of generated image augmentation and the classification accuracy of the network, up to a saturation point.

**Figure 5:**
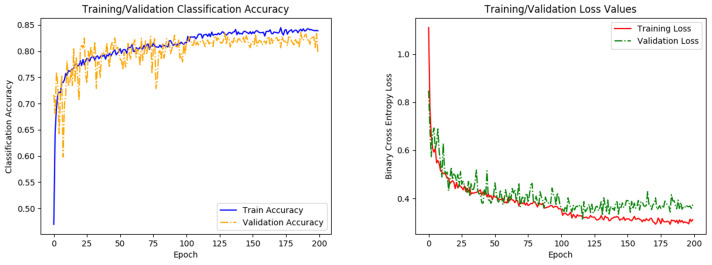
Graphs of network accuracy (**left**) and cross-entropy loss (**right**) for training and validation datasets. A small respective bump/dip in accuracy/loss is observed at 100 epochs, where the learning rate parameter is reduced. Training levels out before the 200 epochs, indicating that the network has finished learning.

All models are trained for 200 epochs (training slows down at this mark), using cross-entropy loss criterion, and stochastic gradient descent optimizer (LR: 0.005, momentum: 0.8, weight decay: 0.0001, batch size: 64), where the learning rate is reduced by half, halfway through training. All configurations are trained with fivefold cross validation, using an 80:20, train:test split. Figure 5 displays the classification accuracy and loss parameters observed over the course of training and is used to check for network overfitting and training efficiency. The following section provides specific details about the dataset used in this work, as well as the results of CNN training using augmented datasets from GAN.

## 4. Results and Discussion

### 4.1. Data

Data for this study come from experiments performed by Dr. Barbara Davis in the laboratory of Dr. Prue Talbot. They are aimed at determining the effects of nicotine exposure on diseased, induced pluripotent stem cells (iPSC) expressing the Huntington’s disease (HD) phenotype. HD is a progressive neurodegenerative disorder that affects motor neurons in the adult brain [54,55]. Nicotine has been shown to have a neuroprotective effect on neurodegenerative diseases such as Parkinson’s disease [56]. The theory of this work is that nicotine may have a similar affect in HD nueronal growth and development.

To test this hypothesis, HD iPSC’s are exposed to nicotine at varying concentrations (control, 10^−4^ M, 10^−5^ M) in vitro over the course of a 48-h culture period. Large-size (2908 × 2908) images of cellular culture dishes are collected at 10× magnification using the Nikon Biostation CT, an an automated incubator–microscope unit. Collected images contain thousands of colony areas at various stages of the developmental process and require preprocessing and manual annotation to be used to train the deep learning networks. These images are preprocessed in accordance with the approach described in Section 3.1. The resulting image dataset is then manually annotated for ground-truth as described in the following section.

### 4.2. Ground-Truth Validation

A breakdown of number of images has been shown in Table 5. Images were sorted based on predetermined, visually distinct features presented in each class that correspond to phenotypic differences. Morphological classes were determined by the experts who collected the data to best reflect the phenotypic characteristics of distinct cell colonies. Ground-truth data was obtained via manual annotation by an expert researcher (A.W.) for the entire dataset. These annotations were validated by two additional researchers (G.P., R.T.) on a random subset of image data to provide consensus and check for variability and subjectivity. The original sorting was confirmed by training a neural network on each of the three annotated data subsets and determining which sorting provided the best testing results on a classwise basis. The rationale for the patch-based sampling approach used for this work is presented in the next section.

### 4.3. Patch-Based Sampling

There are two main reasons why a patch-based sampling approach is used in this work. The first reason is that image classes in this dataset are based on morphological phenotype. These morphologies are determined by their subcolony level texture patterns, and image patches provide a local view of these morphologies. The contiguous nature of colony morphology means that multiple classes can appear in a single colony image; the use of image patches provides the most reliable means of limiting class overlap when performing patch-based classification.

The second reason is that GAN training is a data-intensive process, with the counterproductive goal of increasing dataset size. Therefore, a patch-based sampling method is implemented for the following reasons:

- to increase the apparent training dataset size
- to accommodate efficient network architectures (it is widely recognized that GANs are effective when images are relatively small (≤64 × 64) but are prone to mode collapse with high-resolution images)
- to standardize input size, as image crops vary in dimension
- to model low-level features (i.e., fine-grained textures), which show high variation across image patches for a given class
- to increase general variability via patch sampling, which generally improves training
- to aid in the analytical goal of classifying contiguous, multilabel cell colonies in a patchwise manner using only cellular morphology

To this end, the GAN model is used to generate 64×64 patches of colony images. Image quality is measured using various standardized qualitative and quantitative measurements as described in the following section.

### 4.4. Assessment of Generated Image Quality

**Figure 6:**
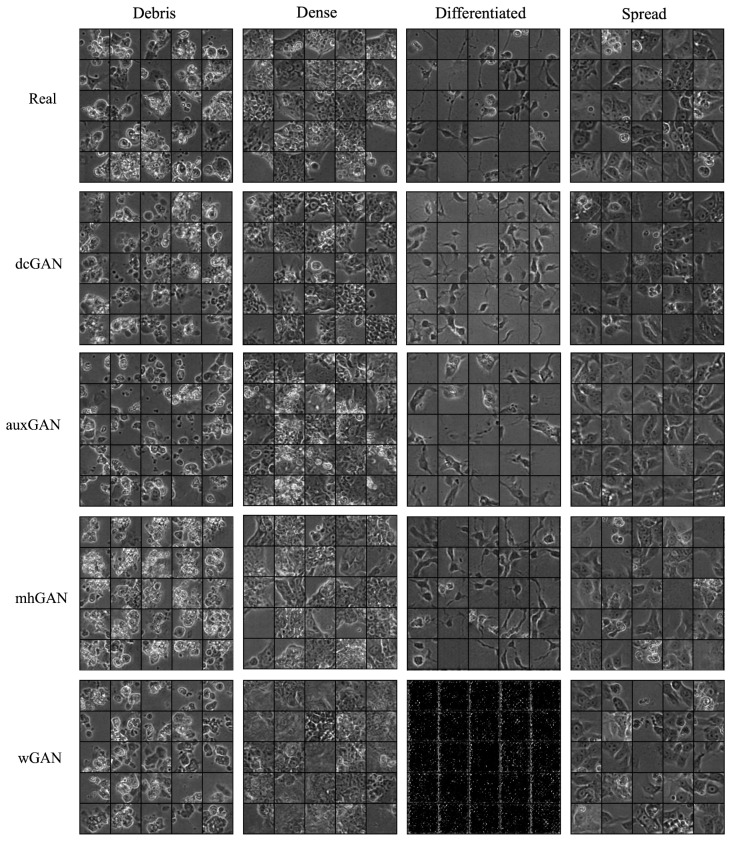
Image patch samples for real and generated images. A comparison of classwise image features displays generally realistic image features indicative of morphological class. However, visual appearance of images provides only a qualitative measure of image quality, where quantitative metrics are necessary to determine image realness.

There are several methods by which generated image quality is measured in this work, including visual appearance, quantitative scores, and efficacy in application for the desired task. Figure 6 displays real and generated image patches across the four morphological classes in the dataset for the various GAN configurations.

Visual assessment of these samples reveals that the generator has been able to capture both the general structure and fine-grained morphological features of the cell colonies, and that these features are distinguishable for the individual cellular phenotypes. Some configurations, such as dcGAN, show greater image variation and visual quality than others, such as auxGAN, especially for the differentiated class. Images generated with the multiclass generator used in auxGAN shows signs of feature entanglement, where image features from one class are present in another. This is because the generator has difficulty separating these features using a single model. Other methods, such as wGAN, fail to generate any realistic images, such as for the differentiated class, where only noisy black images can be seen.

While the generator may be able to convince the trained human eye of its ability to produce realistic images, that does not necessarily mean that it will be able to provide useful information to the learning task. For this, feature-based quality measures are required to determine the level of relative image realness with respect to the real dataset.

#### 4.4.1. Inception Score

Inception score is a quantitative measurement of generated image quality with respect to image-classification-based probability distributions [52,57]. This method combines measurements of generated image realness and variability based on the output predictions of a pretrained inception network. The output is an entropy-based score using the Kullback–Leibler divergence ($D_{KL}$, Equation (12)) between the classwise generated image distribution, *P*, and the overall generated distribution *Q*, where *x* is the discrete probability and *X* is the probability space. In this work, inception score is used to monitor network training, and determine the iteration at which GAN training is optimized. The network representative of this inception score is chosen as the generative model for dataset augmentation. The inception score has a range based on the number of classes in the dataset, for example, from 0 to 1 for binary classification:

(12) $$D_{KL}\left(P||Q\right)=\sum_{x\in X}P\left(x\right)\log\left(\frac{P(x)}{Q(x)}\right)$$

#### 4.4.2. Frécet Inception Distance

The Frécet inception (FID) is another image quality metric that seeks to overcome some of the shortfalls of the inception score by directly comparing generated image distributions to corresponding real image distributions [53]. FID score is calculated using the mean and variance of the output feature maps of the inception network given real and generated image inputs. This method provides a calculation of the image quality with respect to the real images, and is sensitive to noise, blur, and occlusions.

In this work, FID score is used to evaluate the realness of generated image datasets on a classwise basis. Together, the inception score and FID create a comprehensive view of generated image quality and can be used to determine the efficacy of different generator configurations. Observations of image quality made using these metrics are provided in the following section.

### 4.5. GAN Training Visualization

**Figure 7:**
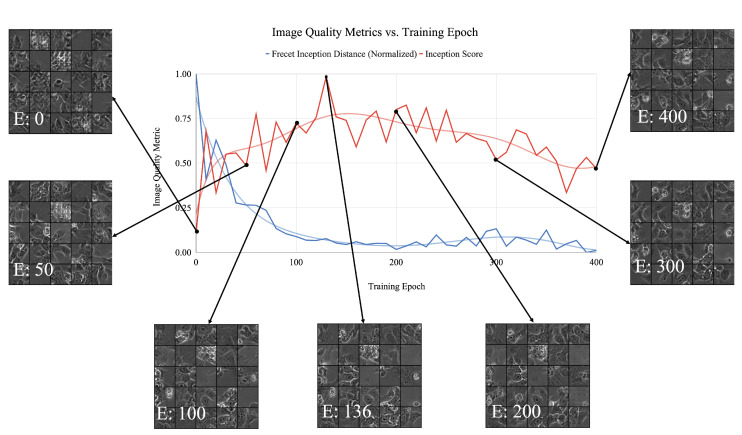
Normalized generator inception score (red) and FID (blue) per training epoch with example images at various intervals for the spread class. Graphs include accompanying trend line. Training epoch numbers are marked by a white ’E’ in the bottom of each image. Agreement between inception score and FID can be seen in terms of their relative minimum and maximum values versus training epoch.

During GAN training, the inception and FID scores for generated image patches is tracked and generated images are gathered at various time points for visual reference (see Figure 7). Many aspects of network learning can be inferred from the observation of these visual references over time. At the start of training (Epoch 1), the generator begins to distinguish between foreground and background, displaying only gray level splotches of indistinguishable colony areas. Images begin to show more features resembling realistic colony patches around the 50-epoch mark, displaying more distinct, lower contrast splotches that also include the high-intensity bright areas indicative of the halos observed in the experimental dataset. These high-level morphological details are seen across the entire dataset and indicate that the network learns high-level features first.

The progression of displayed features indicates that training is stable, but the network has not yet captured the fine-grained texture and gray-level variation that is indicative of the morphological class. These features become more distinct by Epoch 100 as cell boundaries can be seen, and contrast intensifies. Inception score reaches a precipice at the 136-epoch mark (similarly the FID score reaches a valley), and although the GAN continues to produce realistic looking images, it can be noted that the inception score and FID do not improve as training continues past that point.

**Figure 8:**
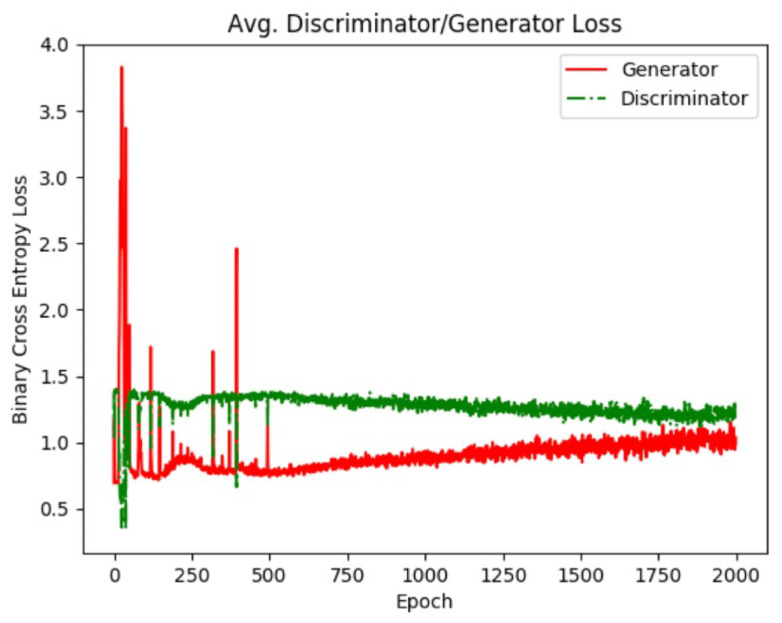
Generator and discriminator loss values for the dense class. As training progresses, the GAN reaches an equilibrium which is when training is considered finished. Using individual GAN models for each image class allows the GAN to be trained for different amounts of time based on the image class.

This plot is useful as a deterministic way of choosing the most realistic generator. Realistic colony features such as clear boundaries between cells, and bright white halos rounded and single cell objects can be seen in the final generated images. Training is considered finished after the GAN loss values have reached equilibrium (Figure 8); however, the training epoch at which the most realistic images are generated is determined using the inception score. The number of epochs required to reach equilibrium is subject to the size of the dataset on a classwise bases, as well as other variations between image data classes. The epoch values at which the inception score for each class is optimal is shown in Table 6, along with their corresponding inception scores at that point. Once the generator is trained for each class, images are generated by simply providing random noise vectors to each of the generator and saving image outputs. In the following section, the ability of different GAN networks to produce realistic images is explored using the defined quality metrics.

### 4.6. GAN Network Comparisons

In this work, multiple GAN configurations are compared to determine the most effective method of image generation, in terms of image realness and variability. The FID (Frécet inception distance) score is used as a quantitative measure of these traits, in a classwise manner, as shown in Table 7, where a lower FID score is better. The different GAN configurations have various potential uses in terms of generative feature learning. For instance, the wGAN configuration is known for preventing mode collapse, whereas the mhGAN is designed to select the most relevant images from the input dataset in terms of the discriminator’s decision output, and for this reason it may be better at modeling multimodal distributions.

**Figure 9:**
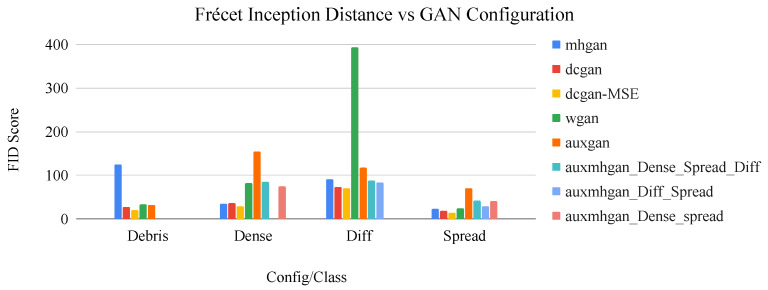
Bar graph of network configuration vs. FID score by image class. The dcGAN+MSE configuration consistently displays the best performance in terms of this metric.

Figure 9 displays these values graphically and shows that the dcGAN+MSE configuration produces the best (lowest) FID score for all classes. The reason for this is that using the entropy loss as a regularizer during GAN training provides additional information to the network about the real image distribution. Subsequently, this allows for the generation of more relevant images in terms of image appearance and variation, as measured by the FID score.

Other configurations, such as wGAN, are not appropriate for this dataset because the trained dcGAN model is not subject to mode collapse, as evident by the variation present in the generated images. For the differentiated class, wGAN is unable to generate relevant images, and produces only image artifacts, hence the relatively high FID score for this class. The reason for this is that wGAN reduces the apparent image variation of this already small class by restricting the feature weights. The mhGAN configurations do not produce higher quality images, in terms of FID score, and takes far longer to train than a standard dcGAN (on the scale of days). mhGAN reduces both the overall number of image samples available to the generator, resulting in lower quality images with less variation. Auxiliary conditional GAN applications (auxGAN, aux-mhGAN) are not as effective at producing the individual image classes using a single model as are individual GAN’s trained on a single class. This is because they are susceptible to feature entanglement, which is the inevitable sharing of input features based on the latent representation of the input data distribution [58].

The ultimate test of the efficacy of the proposed method is to perform generated image augmentation on CNN networks. For this, the dcGAN+MSE generator configuration is used to generate images in line with the temporal classification method as described in Section 3.4. The following section discusses the results of the classification networks trained using the generated dataset augmentation scheme.

### 4.7. Classification Metrics

Classification results for this study are measured using the true positive rate (TPR, Equation (13)) and F1-scores (Equation (14)). TRP, also known as recall, is a measure of the sensitivity of the classifier, where TP is the number of correctly classified positive instances, and FN is the number of incorrectly classified negative instances. F1 is the harmonic mean of precision, which is the TP over the sum of TP and false positives (FP), to recall, and considers the false positives and false negatives equally in its calculation. These metrics provide information about the classifier’s ability to accurately separate positive and negative instances and are used to compare CNN training configurations, as presented in the following section:

(13) $$\mathrm{TPR}=\frac{\mathrm{TP}}{\mathrm{TP}+\mathrm{FN}}$$

(14) $$F1=\frac{\mathrm{TP}}{\mathrm{TP}+\frac{1}{2}\left(\mathrm{FP}+\mathrm{FN}\right)}$$

### 4.8. Dataset Balancing Using Generated Image Augmentation

To test the efficacy of generated image augmentation as a dataset balancing technique, several standard methods of balancing are compared to the generated augmentation method. These techniques include the use of a weighted cross-entropy loss function and an image sampling technique called imbalanced dataset sampler from Ming Yang (repository has over 1400 stars on Github) [59].

Table 8 demonstrates that the generator-balanced dataset configuration outperforms both traditional sampling techniques. This is because sampling and weight balancing inherently detract from the learning ability of one class in favor of another, whereas supplementing the dataset with generated images not only leaves the higher data class intact but also adds useful features to the smaller data classes in the process. Furthermore, these classification scores are improved when balancing data classes in coordination with the temporal classification scheme proposed in this paper.

Exploiting the temporal relationships between image classes increases the average true positive rate of classification is by approximately 2% in the “temporally balanced" training configuration over the “unbalanced" configuration. This increase corresponds to approximately 200 images out of a 10,000-image dataset. In a high-throughput scenario, where millions of images can be collected over the course of a single experiment, this 2% increase can have a substantial effect on the outcome of experimental findings. These hierarchical results are detailed below.

### 4.9. Effect of the Temporal Classification Scheme

The efficacy of using the temporal relationships between image classes to inform network training is explored via a hierarchical CNN classification scheme. Table 9, Table 10 and Table 11 detail the classification metrics in relation to the three stages of temporal classification. First, images are separated into viable (dense/spread/diff.) vs. unviable (debris) classes, which serves to remove colony areas containing dead cells. Areas of debris can be observed within larger colonies of viable cells; however, removing these areas during the first stage of classification negates the possibility of these misclassifications happening in the downstream stages.

It can be seen in Table 9 that the balanced configuration for this first stage improves the true positive rate for the debris class by 1.32% over the unbalanced temporal configuration and 1.36% over the unbalanced four-class configuration (Table 8). The second stage of classification sends the three viable classes to be separated into differentiated vs. dense/spread. This stage is useful in distinguishing between the late-stage adult cells represented by the diff. class, and the early/intermediate stages (dense/spread, respectively). The dense and spread classes are the most closely related in terms of developmental stage; therefore, they have the most similar morphological features, and are misclassified most often given the four-class classification scheme.

The second stage of temporal classification, Table 10, shows an increase in the true positive rate of classification for the differentiated class over the unbalanced temporal configuration by 0.64%. This value represents a smaller increase in comparison to the other classes, which may be due to the relatively low amount of image data for the differentiated class that is available for training GAN and for classification. When looking at the entropy histograms in Figure 3 and overlap values in Table 4, it can be seen that the differentiated class has the lowest overall overlap, which may contribute to the lower improvement in performance.

Additionally, this stage filters out 99% of the dense and spread images, which lends merit to the hypothesis that these classes are most closely related. This stage seeks to use the temporal relationships between early and middle stage colonies and the differentiated colonies as a marker for the classification boundary of these images. Finally, the dense and spread images are sent to the final stage of classification, where they are separated into their individual classes.

The final stage of temporal classification, Table 11, displays an increase in classification rate for the Spread (∼5%) over the unbalanced, four-class configuration, as shown in Table 8. This improvement is shown to be statistically significant (*p*-value = 3.9 × 10^−6^) using Student’s *t*-test. This improvement is also noteworthy given the difficulty of separating these two similar classes.

Table 12 displays the F1-score classification results for the unbalanced configuration and the generator-balanced temporal configuration. This metric shows improvements for the dense, differentiated, and spread classes in the balanced configuration, with an average improvement of approximately 1%, and statistically significant increases in the dense (∼2%) and spread (∼3%) classes, using Student’s *t*-test. These results further illustrate the predictive power of generator augmented, temporally constrained CNN configurations in relation to straightforward classification networks.

Up to this stage of classification, no generated images have been added to the Spread class. As an additional test of the efficacy of generated features, the saturation point of generated image augmentation (i.e., the point at which the accuracy no longer increases with increasing number of generated images) is empirically determined using the dense and spread image classes.

### 4.10. Saturation Point of Generated Image Augmentation

In addition to the problem of imbalanced datasets, dataset limitations represent another problem in the field biological image classification. These limitations are due to the difficult nature of biological experimentation, in terms of time, money, and experimental yield. Often, image datasets are relatively small in terms of the amount of data needed to efficiently train a neural network. Therefore, generated image augmentation could be a viable means of increasing the apparent dataset size and available features for deep learning applications.

To test this theory, generated images are added to the dense and spread classes to train a two-class CNN until the classification accuracy no longer improves with increasing number of generated images. This point represents the saturation point of the network, where the generated images are no longer providing more useful features to the model. This test also serves as a method of determining the effectiveness and practicality of dataset augmentation using generated image features because it directly compares the number of added images to the improvement in performance.

**Figure 10:**
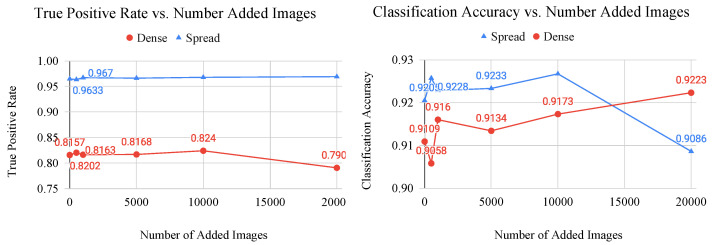
Graphs of classification metric (left: true postitive rate, right: classification accuracy) vs. number of added generated images for the dense and spread classes. These graphs are used to determine the saturation point of a CNN, which is where the generated images no longer provide useful features to the model.

Figure 10 details the results of these experiments. The graphs of generated image augmentation show a positive linear relationship for both the dense and spread classes (in terms of TPR and Classification Acc.) until about the 10,000 image mark, where improvement falls. This could be indicative of the network overfitting on generated images and failing to learn the features of real images. While these improvements are small in comparison to those of the dataset balancing experiments, they confirm that limited dataset supplementation is a viable avenue for generated image augmentation.

## 5. Conclusions

The temporally constrained, generative dataset augmentation scheme employed in this paper represents an improvement in the performance of deep learning algorithms for classification tasks involving limited and imbalanced biological image datasets. Known GAN methods are compared to generate images, and then image–class relationships are employed to design a temporal classifier. The method shows classification improvements for all image classes, as measured by true positive rate and F1-score, without sacrificing the performance of any class. Moreover, this work highlights the importance of exploiting domain knowledge in similar tasks, which, in this case, comes in the form of the temporal relationships between image classes. While deep learning has become the gold standard in terms of image feature representation and learning, it is not a substitute for prior biological knowledge. To this end, one item of future work will involve the expansion of this work to include video data to incorporate cellular dynamics. Overall, the combination of domain knowledge with deep learning is paramount for the effective modeling of biological image features, and it allows for the synergistic design of deep-learning-based algorithms, and experimental data collection. 

## Figures and Tables

**Table 1:** Morphological class descriptions and corresponding biological implications.

Class	Morphological Description	Implication
Debris	Individual cells or aggregates cells showing circular morphology with high intensity white ‘halo’ marking distinct boundaries	Distressed, dead (apoptotic/necrotic) cells that float on top of colony indicating negative response to experimental conditions
Dense	Homogeneous aggregates of small cells with indiscernible cell boundaries, no clear nucleus	Induced pluripotent stem cell colonies that maintain undifferentiated status under current conditions
Spread	Homogeneous aggregates of large cells with discernible cell boundaries, clear nuclei, large protrusions	Down stream lineage intermediates or progenitor cells
Differentiated	Individual cells or spaced out aggregates of cells with distinct, dark cell bodies, high-intensity white boundaries, and dark axon like protrusions.	Differentiated neurons or neuronlike downstream lineages

**Table 2:** GAN Network Architecture: The input to the GAN generator (a) is a latent vector of length 100 multiplied which is processed through multiple convolutional (C2d) and upsampling (Up) layers. The output of the generator is a hyperbolic tangent (Tanh). The output of the discriminator (b) goes to a fully connected (FC) layer followed by a sigmoid function (Sig).

Generator			Discriminator		
**Module**	**Size**	**Maps**	**Module**	**Size**	**Maps**
Linear	1×100/16	1/512	C2d	64/32	1/64
Up	16/36	512/512	C2d	32/16	64/128
C2d	36/36	512/512	C2d	16/8	128/256
Up	36/64	512/512	C2d	8/4	256/512
C2d	64/64	512/256	FC	8192	512
C2d	64/64	256/1	Sig(·)	1/1	-/-
Tanh(·)	64/64	1/1			

**Table 3:** GAN training hyperparameters were empirically determined to optimize network training efficiency.

Training Hyperparameters	
Parameter	Value
Learning Rate-Adam	0.002
β_1_—Adam	0.5
β_2_—Adam	0.999
Max feature maps—Discriminator	512
Max feature maps—Generator	512

**Table 4:** Overlap percentage for image entropy histograms across five trials, for which the entropy values of 50,000 random real and generated image patches each are plotted and the overlap is calculated. Variation in values is caused by randomly generated image patches that contain variability within the image. These values can be used to determine how well the generator has been able to model image features and can be correlated with the performance of downstream dataset augmentation tasks.

Image Class	Overlap Percentage—Mean (std.)
Debris	0.6182 (0.0026)
Dense	0.7066 (0.0033)
Diff	0.3936 (0.0018)
Spread	0.3999 (0.0011)

**Table 5:** Data breakdown for four morphological classes. Class imbalances observed here are a factor of the natural growth and differentiation cycle of the cells.

Class	# Samples
Debris	3587
Dense	3934
Diff	656
Spread	10,506
Total	18,683

**Table 6:** Epoch values and corresponding inception scores at which the GAN generator is determined to be optimally trained, based on the plot of inception score vs. training epoch. Each GAN is trained on an individual class, and, therefore, requires a different level of training based on the number of images in each class, as well as complexity of features and other variables. The optimal GAN is used to generate images for each class to be used for dataset augmentation.

Image Class	Optimal Generator Epoch	Inception Score
Debris	116	2.60
Dense	444	2.32
Diff	225	2.38
Spread	136	2.57

**Table 7:** Frécet inception distance (FID) scores for each GAN configuration by image class. An x indicates where the GAN was not trained to generate the specific image class.

Config./Class/FID	Debris	Dense	Diff.	Spread	Average
dcGAN	27.73	36.32	72.77	18.51	38.83
dcGAN + MSE	19.5	29.5	70.7	13.67	33.34
wGAN	33.85	81.45	393.94	24.05	133.32
auxGAN	31.62	155.13	117.92	69.86	93.63
mhGAN	125.22	35.03	90.63	23.05	68.48
aux-mhGAN (Dense, Diff, Spread)	x	84.93	88.7	41.53	71.72
aux-mhGAN (Dense, Spread)	x	x	83.37	29.55	57.54
aux-mhGAN (Diff, Spread)	x	74.0	x	41.05	56.46

**Table 8:** Classwise true positive rate for four-class CNN with and without dataset balancing. Several variations of balancing are used here, the most effective of which is supplementation using generated images in line with the temporal training configuration proposed in this paper. The *p*-value indicated by the * is calculated using Student’s *t*-test.

Configuration/Class/TPR (Std.)	Debris	Dense	Diff.	Spread	Average
Unbalanced	0.9141 (0.0144)	0.8093 (0.0211)	0.8807 (0.0342)	0.9144 (0.0093) ^*^	0.8789
Sampler Balanced	0.8570 (0.0184)	0.9300 (0.0189)	0.9274 (0.0219)	0.8410 (0.0073)	0.8888
Weight Balanced	0.9030 (0.0312)	0.8065 (0.0249)	0.8439 (0.0715)	0.9300 (0.0290)	0.8708
Generator Balanced	0.9105 (0.0206)	0.7940 (0.0116)	0.8999 (0.0247)	0.9172 (0.0124)	0.8804
Temporally Balanced	0.9277 (0.0148)	0.8157 (0.0142)	0.8856 (0.0289)	0.9646 (0.0040) ^*^	0.8984

**Table 9:** Hierarchical tier 1: true positive rate for temporal combination of viable cell classes vs. debris cells. This stage acts as a filtration step to remove unviable and unhealthy colony areas.

Configuration/Class/TPR (std.)	Debris	Dense/Diff./Spread	Average
Unbalanced	0.9145 (0.0097)	0.9570 (0.0058)	0.9357
Generator Balanced	0.9277 (0.0148)	0.9545 (0.0053)	0.9411

**Table 10:** Hierarchical tier 2: true positive rate for separation of dense/spread classes from differentiated. This tier serves to remove the mature cell colonies from the early and intermediate stage classes. The dense/spread classes have the highest level of misclassification, due to their relative proximity in terms of the downstream differentiation process, and subsequent similarity in texture features.

Configuration/Class/TPR (std.)	Diff.	Dense/Spread	Average
Unbalanced	0.8792 (0.0255)	0.9941 (0.0007)	0.9367
Generator Balanced	0.8856 (0.0289)	0.9935 (0.0007)	0.9396

**Table 11:** Hierarchical tier 3: true positive rate for classification of dense vs. spread. The balancing of the dense class, using generated images, in relation to the Spread class shows slight improvement over the unbalanced configuration, and marked improvement over the four-class configuration.

Configuration/Class/TPR (std.)	Dense	Spread	Average
Unbalanced	0.8187 (0.0140)	0.9624 (0.0035)	0.8906
Generator Balanced	0.8157 (0.0142)	0.9646 (0.0040)	0.8902

**Table 12:** F1-score for unbalanced and generator-balanced temporal training configurations show an overall improvement for the balanced configuration on average, as well as statistically significant increases in the dense and spread classes. The *p*-value indicated by the * is calculated using Student’s *t*-test.

Configuration/Class/F1 (std.)	Debris	Dense	Diff.	Spread	Average
Unbalanced	0.8732 (0.0059)	0.8430 (0.0082) *	0.8580 (0.0164)	0.9119 (0.0036) **	0.8715
Generator-Balanced	0.8599 (0.0099)	0.8599 (0.0050) *	0.8714 (0.0009)	0.9433 (0.0030) **	0.8836

## Data Availability

Data available upon request.
